# Phylodynamic Profile of HIV-1 Subtype B, CRF01_AE and the Recently Emerging CRF51_01B among Men Who Have Sex with Men (MSM) in Singapore

**DOI:** 10.1371/journal.pone.0080884

**Published:** 2013-12-02

**Authors:** Kim Tien Ng, Kah Ying Ng, Wei Xin Khong, Kuan Kiat Chew, Palvinder Kaur Singh, Joe Kwan Yap, Mei Ting Tan, Yee Sin Leo, Oliver Laeyendecker, Thomas C. Quinn, Adeeba Kamarulzaman, Kok Keng Tee, Oon Tek Ng

**Affiliations:** 1 Centre of Excellence for Research in AIDS (CERiA), Department of Medicine, Faculty of Medicine, University of Malaya, Kuala Lumpur, Malaysia; 2 Institute of Infectious Disease and Epidemiology, Communicable Disease Centre, Tan Tock Seng Hospital, Singapore; 3 Division of Infectious Diseases, Johns Hopkins School of Medicine, Baltimore, Maryland United States of America; 4 Laboratory of Immunoregulation, Division of Intramural Research, National Institute of Allergy and Infectious Disease, National Institutes of Health, Baltimore, Maryland United States of America; University of Florida, United States of America

## Abstract

HIV-1 subtype B and CRF01_AE are the predominant infecting subtypes among men who have sex with men (MSM) in Singapore. The genetic history, population dynamics and pattern of transmission networks of these genotypes remain largely unknown. We delineated the phylodynamic profiles of HIV-1 subtype B, CRF01_AE and the recently characterized CRF51_01B strains circulating among the MSM population in Singapore. A total of 105 (49.5%) newly-diagnosed treatment-naïve MSM were recruited between February 2008 and August 2009. Phylogenetic reconstructions of the protease gene (HXB2: 2239 – 2629), gp120 (HXB2: 6942 – 7577) and gp41 (HXB2: 7803 – 8276) of the *env* gene uncovered five monophyletic transmission networks (two each within subtype B and CRF01_AE and one within CRF51_01B lineages) of different sizes (involving 3 – 23 MSM subjects, supported by posterior probability measure of 1.0). Bayesian coalescent analysis estimated that the emergence and dissemination of multiple sub-epidemic networks occurred between 1995 and 2005, driven largely by subtype B and later followed by CRF01_AE. Exponential increase in effective population size for both subtype B and CRF01_AE occurred between 2002 to 2007 and 2005 to 2007, respectively. Genealogical estimates suggested that the novel CRF51_01B lineages were probably generated through series of recombination events involving CRF01_AE and multiple subtype B ancestors. Our study provides the first insight on the phylodynamic profiles of HIV-1 subtype B, CRF01_AE and CRF51_01B viral strains circulating among MSM in Singapore.

## Introduction

Human immunodeficiency virus type 1 (HIV-1)is known for its extreme genetic diversity, owing to its high mutation rates, high viral turnovers and the persistent nature of its infection [1]. In addition to the 9 major subtypes (A - D, F - H, J and K) of the HIV-1 group M, frequent intersubtype recombinations have further diversified the virus into at least 61 known circulating recombinant forms (CRFs) and many unique recombinant forms (URF) [2]. While HIV-1 prevalence among the general population in low- and middle-income countries has exhibited a decreasing trend in recent years, men who have sex with men (MSM) continue to be disproportionately affected by HIV-1 infection, with approximately 19 times odds of being infected with HIV-1 [3]. Recent surveillance reports have shown significant increase in the prevalence of HIV-1 infections among MSM in several Asian countries [4]-[6].

In 2002, Kalish et al. [7] observed a pattern of HIV-1 subtype segregation by the main transmission risk groups in Singapore, namely MSM and heterosexual, that were commonly infected with HIV-1 subtype B and CRF01_AE, respectively. A more recent study further confirmed that HIV-1 subtype B and CRF01_AE continue to be the predominant genotypes circulating in Singapore, with a higher CRF01_AE prevalence among MSM [8]. In addition, Ng et al. [9] reported the emergence of a novel HIV-1 CRF51_01B lineage among MSM in Singapore which is genetically distinct from other previously established subtypes or recombinants. Despite the significant clinical and epidemiological impact attributed to HIV-1 subtype B, CRF01_AE and CRF51_01B in Singapore [10], the genetic history, population dynamics and pattern of transmission networks of these genotypes remains largely unexplored. Through better understanding of the origin and evolutionary behaviours of these genotypes and sexual network analysis, effective preventive measures can be customized to prevent HIV-1 transmission among MSM [11], [12]. In the present study, based on the HIV-1 protease and *env* (gp120 and gp41) genes, we attempt to delineate the genetic history and the phylodynamic profiles of HIV-1 subtype B and CRF01_AE, as well as the little known CRF51_01B lineages isolated from the MSM population in Singapore, by using a suite of phylogenetic tools that involve maximum likelihood and Bayesian coalescence approaches.

## Materials and Methods

### Ethics Statement

The study was approved by the National Healthcare Group Ethics Committee. Written informed consent was obtained from all subjects prior to sample collection.

### Study Subjects and Specimens

A total of 212 treatment-naïve HIV-1 positive subjects were interviewed and recruited from the Singapore Communicable Disease Centre (CDC) outpatient clinic between February 2008 and August 2009. Demographic information, namely age, gender and ethnicity, as well as transmission risk factors were obtained, followed by collection of plasma specimens that were serologically tested and confirmed to be HIV-1 positive.

### Phylodynamic Inference and Divergence Times of Transmission Clusters

As previously described by Ng et al [8], HIV-1 RNA was extracted, reverse-transcribed, followed by nested PCR amplification and population sequencing of the protease gene (HXB2: 2239 – 2629, 390 bp), gp120 (HXB2: 6942 – 7577, 636 bp) and gp41 (HXB2: 7803 – 8276, 474 bp) of the *env* gene. HIV-1 genetic subtype and potential transmission networks in this dataset were first deduced from neighbour-joining tree of each genomic region [13] using MEGA version 5.05 based on Kimura-2 parameter model [14]. The reliability of each transmission networks were further evaluated by a more robust maximum likelihood phylogenies, heuristically inferred using subtree pruning and regrafting, as well as nearest neighbour interchange algorithms implemented in PAUP version 4.0 [15]. The statistical significance of the branching orders was evaluated by bootstrap analysis of 1000 replicates. The most appropriate nucleotide substitution model was determined using FindModel, a web implementation of Modeltest available at the HIV database (http://www.lanl.gov.com). In addition, Bayesian maximum clade credibility (MCC) trees were constructed using BEAST 1.7 [16] to determine the posterior probability values for each network in subtype B, CRF01_AE and CRF51_01B. As previously described elsewhere, transmission network is defined as a network consisting of at least 3 isolates from different individuals [17] of the same geographical (i.e country) origin [18], and a phylogenetic clade supported by bootstrap value of more than 90% and Bayesian posterior probability value of 1.0 at the tree node [17], [19], [20].

The time of most recent common ancestor (tMRCA) of the respective transmission networks observed in each genomic region were estimated by the Bayesian coalescent-based relaxed molecular clock model, conducted in BEAST 1.7.4[16]. The uncorrelated lognormal model [21] nested in general time-reversible (GTR) nucleotide substitution model [22] with a proportion of invariant sites and four rate categories of gamma-distribution model of among site rate heterogeneity [23] was employed to estimate HIV-1 phylogenies, nucleotide substitution rates and to date the tMRCAs of each transmission networks. Different parametric demographic models namely constant population size, exponential and logistic growth model as well as a nonparametric Bayesian skyline plot (BSP) was tested, with the best fits coalescent model selected by means of a Bayes factor (BF), determined by Tracer version 1.5 (http://tree.bio.ed.ac.uk/software/tracer), using marginal likelihood [24]. Bayes factors can be interpreted as follows: 2InBF < 2 no evidence; 2 – 6 weak evidence; 6 – 10 strong evidence; > 10 very strong evidence [25]. The Markov chain Monte Carlo (MCMC) analysis was computed for 50 million states sampled every 10,000 states and output was assessed for convergence by means of effective sampling size (ESS) after a 10% burn-in using Tracer. To minimize the effects of standard errors, only traces with ESS of at least 200 were accepted. Prior to the inference of tMRCA for each transmission network [26], tMRCA for subtype B′ of Thai origin (isolates 93CNRL42, 96M145, 96TH_NP1538, 96M081, 99TH_C1416, 99MMmSTD101, 00TH_C3198, 01CNHN24, 02CNHNsc11, 02CNHNsmx2 and 02CNHNsq4), which were thought to be the descendent of the ancestral subtype B lineages that emerged around 1980 to 1991 [27], [28], were co-estimated and checked for aberration with the current tMRCA estimations.

Given the fact that each HIV-1 sequence in this time-scaled phylogeny represents a different subject, the branches linking two sequences through their most recent common ancestor (MRCA) represents one transmission event. Consequently, the distance between the MRCA and the previous node deduced the upper bound of the year between transmission events. To obtain a more representative picture on the magnitude of transmission in this cohort, the transmission events (in years) of these networks were estimated.

### Sequences

Partial sequences of the HIV-1 protease gene, gp120 and gp41 of *env* gene analyzed in this study have been deposited in GenBank under the accession numbers listed in **Table S1**.

## Results

Between February 2008 and August 2009, 105 treatment-naïve HIV-1 positive MSM were recruited. In the present study, 89 (37 subtype B, 40 CRF01_AE and 12 CRF51_01B) interpretable protease sequences, 54 (16 subtype B, 29 CRF01_AE and 9 CRF51_01B) gp120 and 96 (43 subtype B, 42 CRF01_AE and 11 CRF51_01B) gp41 of *env* gene sequences were used for analysis. In addition to unique transmission lineages that do not cluster (data not shown), substantial clustering patterns (protease region: 64.0%; 57/89, gp120 region: 70.4%; 38/54 and gp41 region: 62.5%; 60/96) were observed.

**Figure 1:**
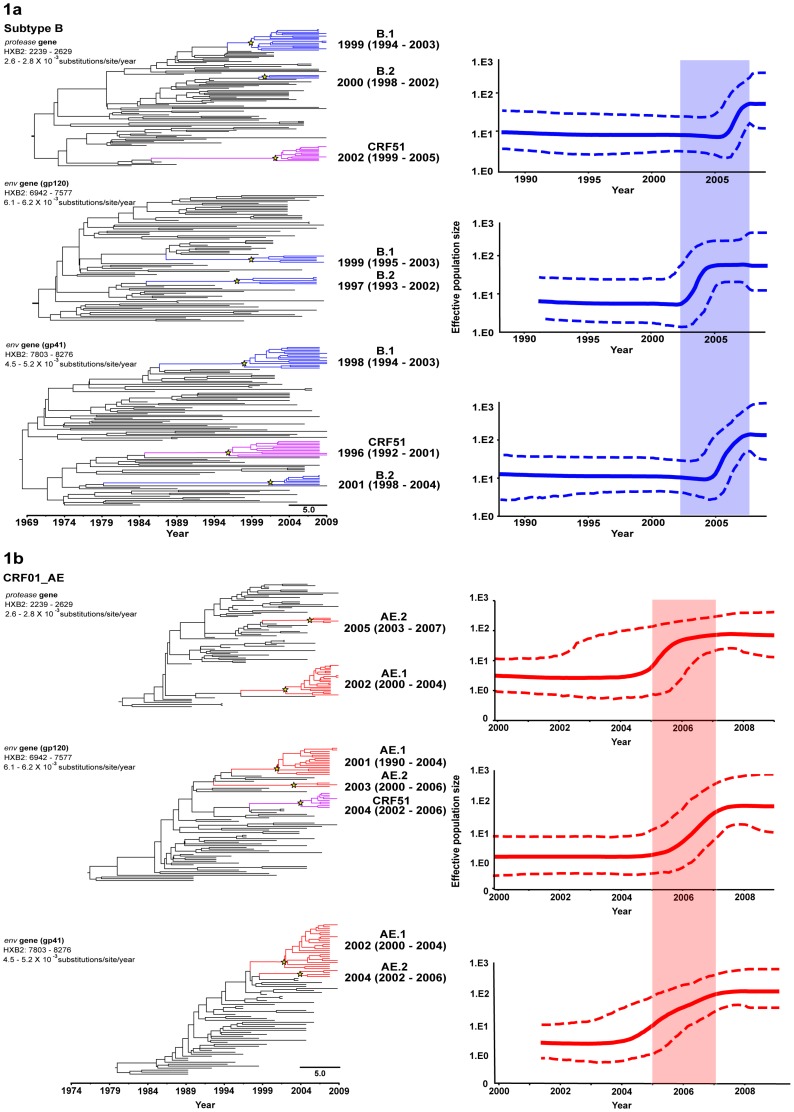
Phylodynamic profile of HIV-1 subtype B, CRF01_AE and CRF51_01B among men who have sex with men (MSM) in Singapore. For clarity, only monophyletic transmission networks are shown. Bayesian’s MCC trees of protease gene (HXB2: 2239 – 2629, 390bp), gp120 (HXB2: 6942 – 7577, 636bp) and gp41 (HXB2: 7803 – 8276, 474bp) of *env* gene from Singapore MSM sequence data collected between February 2008 and August 2009 are shown, with Singapore HIV-1 subtype B branches in blue (**1a**), CRF01_AE in red (**1b**), CRF51_01B in purple and reference sequences in black branches. Of note, CRF51_01B is a recombinant involving subtype B in protease and gp41 of *env* gene, and CRF01_AE in gp120. The Bayesian coalescent-based relaxed molecular clock model was performed in BEAST 1.7, with uncorrelated lognormal model coupled with general time-reversible (GTR) nucleotide substitution model and a proportion of invariant sites. The Markov chain Monte Carlo (MCMC) analysis was computed for 50 million states sampled every 10,000 states and output was assessed for convergence by means of effective sampling size (ESS) after a 10% burn-in. Transmission networks are defined based on strong statistical inferences generated at the internal nodes of the maximum likelihood and MCC tree reconstructions (bootstrap values of more than 90% and posterior probability of 1, respectively). The mean tMRCA and 95% highest posterior distribution (HPD) for each network are indicated in parentheses. The scale bar indicates the time in year. Bayesian skyline plots (BSPs) generated based on the time-stamped protease gene (HXB2: 2239 – 2629, 390bp), gp120 of *env* gene (HXB2: 6942 – 7577, 636bp) and gp41 of *env* gene (HXB2: 7803 – 8276, 474bp) from the MSM population in Singapore are shown. The origin and changes in effective population size through time for subtype B and CRF01_AE in the country were estimated. The 95% HPD of the effective population size is indicated in dashed lines.

Based on the significant statistical supports generated at the internal tree nodes of maximum likelihood and Bayesian’s maximum clade credibility (MCC) tree analysis for the three genetic regions, phylogenetic reconstruction uncovered four monophyletic transmission networks, two each within subtype B lineages, denoted B.1, with 5 – 17 MSM subjects and B.2, with 3 – 7 MSM subjects **(** **Fig. 1a** **)** and CRF01_AE lineage, denoted AE.1, with 16 – 23 MSM subjects and AE.2, with 3 – 4 MSM subjects **(** **Fig. 1b** **)**. In addition, one transmission network was observed within CRF51_01B lineages, denoted CRF51, with 9 – 12 MSM subjects **(** **Fig. 1a and 1b** **).** CRF51_01B is a recently described recombinant, involving subtype B in protease and gp41, and CRF01_AE in gp120 of *env* gene [8], [9]. Chi-square analysis indicated that such clustering pattern have no significant association with ethnicity (*P* = .69), age (*P* = .86), viral load (*P* = .097) and CD4 count (*P* = 0.81)

Analyses of these three genomic regions under a relaxed clock (uncorrelated lognormal) and constant coalescent model revealed a mean evolutionary rate of 2.6 – 2.8 (95%credible region: 2.1 – 3.5)×10^−3^ substitutions/site/year in protease region, 6.1 – 6.2 (5.0 – 7.6)×10^−3^ substitutions/site/year in gp120 region and 4.5 – 5.2 (3.8 – 6.4)×10^−3^ substitutions/site/year in gp41 region, overlaps with estimates reported elsewhere [29]. Based on these evolutionary parameters and estimates, the most recent common ancestor (tMRCA) of each transmission network was estimated. The transmission networks of subtype B (B.1 and B.2) mainly surged around late 1990s to early 2000s with the oldest network most likely emerging as early as 1993 **(** **Fig. 1a** **)**, while CRF01_AE transmission networks (AE.1 and AE.2) diverged in early 2000s **(** **Fig. 1b** **)**. Divergence times estimated for each subtype B and CRF01_AE networks were concordant between genetic regions. In contrast, the tMRCAs for the recently isolated CRF51_01B were estimated around 2002 (1999 – 2005), 2004 (2002 – 2006), and 1996 (1992 – 2001) in the protease gene, gp120 and gp41 of *env* gene, respectively. In addition, the tMRCA for reference subtype B′ isolates was estimated at around the late 1980s in all three genetic regions and in congruence with the findings from other studies [27], [28], [30].

To illustrate the past population dynamics of HIV-1 circulating among the Singapore MSM, temporal changes in effective population size over the last two decades for subtype B and CRF01_AE were estimated. Bayesian skyline plot indicated the initial growth phase for subtype B lineages started in the late 1980s, followed by an exponential growth started between 2002 and 2007, and reached its peak from 2007 onwards **(** **Fig. 1a** **)**. For CRF01_AE lineages, the initial growth phase radiated in the early 2000s, and later undergone regular increase around 2005 to 2007, and reached sustained growth thereafter **(** **Fig. 1b** **)**.

To estimate the time between transmission events within networks, each HIV-1 sequences in the time-scaled phylogeny represents a different patient, and distance between two adjacent tMRCAs denotes the upper boundary of the time between 2 transmission events. Estimation of the divergence times for each node revealed that the median interval between transmission events within these networks were short: 0.9 years (0.1 – 4.7) in subtype B, 0.75 years (0.1 – 1.7) in CRF01_AE and 0.65 years (0.1 – 2.2) in CRF51_01B.

## Discussion

Although HIV-1 transmission is increasing among the MSM population in Singapore and other countries in the region, a comprehensive analysis on the viral diversity, growth and temporal dynamics in this high-risk group remains scarce particularly in Southeast Asia. To our knowledge, this is the first study in Singapore investigating the transmission behaviour and evolutionary history of the co-dominating HIV-1 subtype B, CRF01_AE and newly emerged CRF51_01B among the MSM population based on the time-stamped sequence data collected between 2008 and 2009. Here, we highlight the possible key factors that might have association with the pattern of HIV-1 infections among MSM in Singapore, namely the establishment of transmission networks, active spread of HIV-1 through MSM network and lastly, the emergence of CRF51_01B.

The HIV-1 *pol* gene has proven informative and important in evaluating and defining transmission networks within a population of interest [31]–[33]. In the present study, gp120 and gp41 of *env* gene were co-analyzed. By definition, transmission networks are phylogenetic networks consisting of 3 or more nucleotide sequences from different individuals that are supported by strong statistical evidence, namely bootstrap value of more than 90% and Bayesian posterior probability of 1.0. Based on these inclusion criteria, significant clustering pattern was observed. Bayesian coalescent analysis estimated that the tMRCAs for all transmission networks originated between 1995 and 2005, with subtype B radiated around late 1990s, followed closely by CRF01_AE that emerged in early 2000s. The short median interval (less than 1 year) between transmission events within these transmission networks indicate an ongoing active HIV-1 transmission and suggest possible epidemic expansion due to high-risk sexual behaviour among the MSM population [5], [34], [35]. The identification of viral transmission clusters coupled with the estimated tMRCAs suggest that HIV-1 subtype B and CRF01_AE lineages were introduced into the MSM population in Singapore through at least 5 known sub-epidemics, which occurred between 1995 and 2005. Of note, similar findings in the same time frame were recently reported in neighbouring country, Malaysia [36] and in Western Europe [20], [34], [35], indicating that the emergence of transmission networks was likely caused by the increasing exposure to high-risk behaviours among MSM. Taken together, these findings suggest that most of HIV-1 infections among MSM in Singapore were likely to be linked to local networks. In addition, Brenner et al [37] had illustrated the direct correlation between networks size, transmission events and expansion rate, revealing the importance of understanding these transmission networks during assessment of ongoing epidemic or expanding outbreak. In the present study, such correlation was not observed, possibly due to the sample size of the study, sampled between February 2008 and August 2009 from one clinical centre. In order to have a clearer representation, an in-depth sampling approach spanning a longer recruitment period and involving all clinical centres in Singapore should be employed.

The first case of HIV-1 infection in Singapore was first identified in 1985, with the first case of AIDS reported in September 1986 [38]. Of note, Bayesian skyline plot for subtype B lineage indicated the initial growth phase started in late 1980s, corroborating with epidemiological data that HIV-1 might have been introduced into Singapore MSM population around the mid 1980s. Genetic data also demonstrated the evolutionary history of HIV-1 subtype B and CRF01_AE lineages spanning a period of almost two decades. Collectively, the Bayesian skyline plot showed an epidemic expansion from early to late 2000s, with a 10-fold exponential growth in effective population size of subtype B and CRF01_AE observed between 2002 – 2007 and 2005 – 2007, respectively.

Interestingly, the genealogical analysis of the recently described CRF51_01B revealed tMRCA estimates of 2002 (1999 – 2005), 2004 (2002 – 2006), and 1996 (1992 – 2001) in the protease, gp120 and gp41 of *env* gene, respectively. Such tMRCA estimates may suggest the following: a) the significantly older gp41 region indicates that there were at least two recombination events involving subtype B lineages of different evolutionary histories i.e. distinct divergence times, and b) the possible existence of a yet identified intermediate recombinant form (a founding precursor for CRF51_01B) that was circulating at low level or have since gone extinct.

From the perspective of public health, sexual networks within the MSM population could serve as an important factor in onward HIV-1 transmission, thus it functions as the core entry point for the delivery of intervention strategies. However, the effectiveness and the success rate of any approach largely depends on factors such as ethnicity, race and most importantly, the mindset of general population towards MSM population [39], that may decrease the health-seeking behaviours among MSM [40]–[42]. In this context, educating both general population and MSM population on disease transmission and antiretroviral-based prevention strategy is imperative to prevent onward HIV-1 transmission.

Due to social stigmatization, information on epidemic linkages among the subjects that may assist the transmission network estimation such as numbers of sexual partners, their sexual practices (receptive or insertive anal intercourse), ethnic preference, the use of condoms, location of infection and travel history are not readily revealed by the subjects. However, in this study, we show that the presence of possible predisposing epidemic linkages in a population (particularly those with limited epidemiological data) can be estimated largely based on the genetic information, highlighting the importance (and the ability) of viral genetic information in assessing the epidemic linkages and to study the viral phylodynamic. Since the present study only involved subject recruited between 2008 and 2009, we believe a study spanning a longer period may improve the resolution of HIV-1 genomic diversity and transmission dynamics within the MSM networks in Singapore.

## Supporting Information

Table S1 **Accession numbers and the partial sequences of the HIV-1 protease gene, gp120 and gp41 of** ***env*** **gene analyzed in this study.**
(TIF)
