# Supplementary figures and images for: Phylodynamic Profile of HIV-1 Subtype B, CRF01_AE and the Recently Emerging CRF51_01B among Men Who Have Sex with Men (MSM) in Singapore
Source: PLoS One. 2013 Dec 2;8(12):e80884. doi: 10.1371/journal.pone.0080884 (PMC3846621; doi:10.1371/journal.pone.0080884)

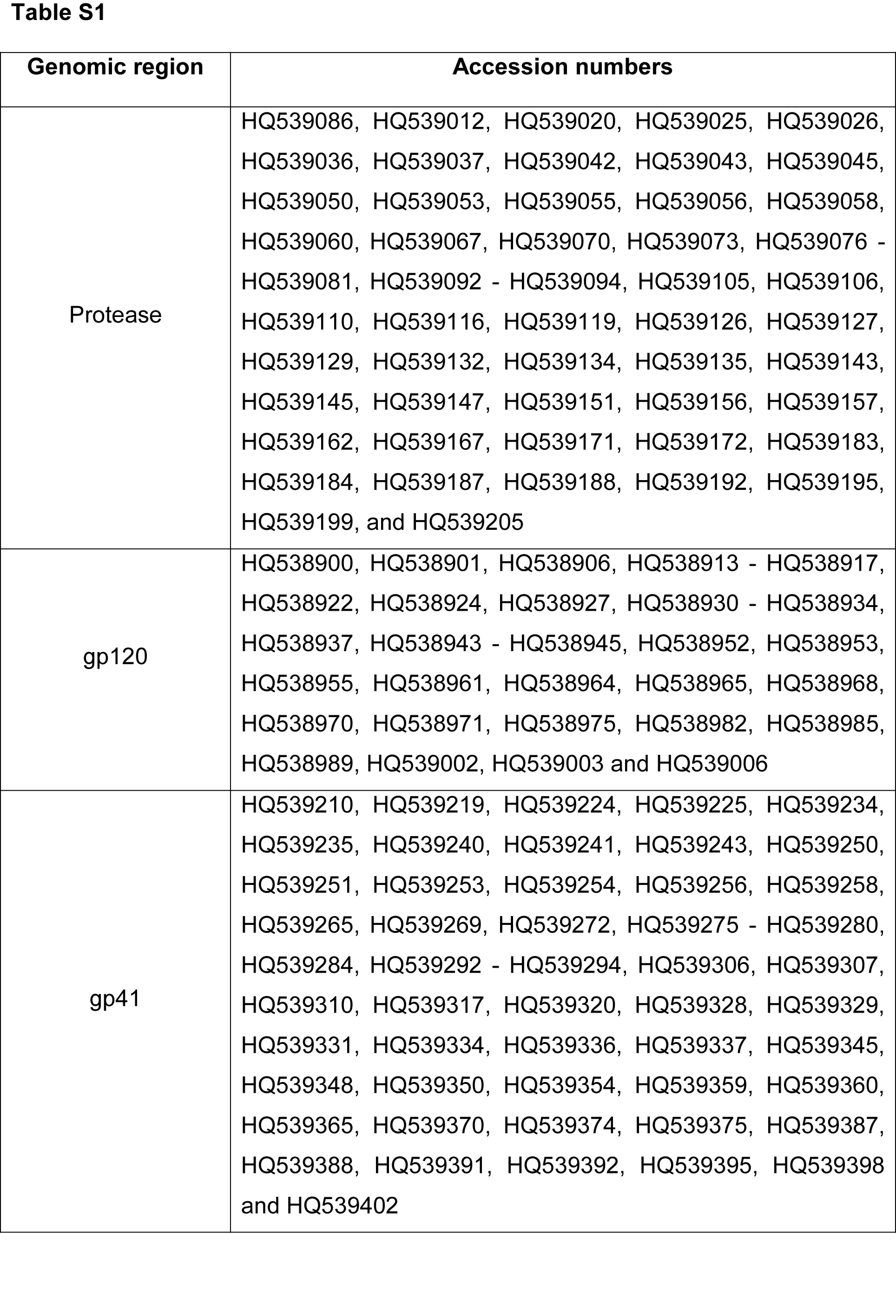

Supplement: Table S1 — Accession numbers and the partial sequences of the HIV-1 protease gene, gp120 and gp41 of env gene analyzed in this study. (TIF) [file pone.0080884.s001.tif]
